# Coping in crisis: The role of sense of coherence, life satisfaction, and resilience in the relationship between depression, social support, fear of COVID-19, and perceived vulnerability to disease among nurses in South Africa

**DOI:** 10.1177/13591053241279000

**Published:** 2024-09-30

**Authors:** Bronwyne Coetzee, Phillipa Haine, Martin Kidd, Lindokuhle Shongwe, Marnus Janse Van Vuuren, Ashraf Kagee

**Affiliations:** 1 Stellenbosch University, South Africa

**Keywords:** depression, nurses, social support, sense of coherence, satisfaction with life, South Africa

## Abstract

In the context of the formidable challenges posed by the COVID-19 pandemic, healthcare professionals coped in various ways. This cross-sectional survey study sought to examine the protective role of satisfaction with life, sense of coherence, and resilience in the relationship between depression, social support, fear of COVID-19, and perceived vulnerability to disease among nurses in South Africa. Participants were a convenience sample of nurses (*n* = 264) working at four South African hospitals in the Western Cape. Data were collected by means of an electronic survey and analysed using structural equation modelling. Participants completed a comprehensive battery of psychological measures. We found that while higher levels of fear of COVID-19 robustly predicted depressive symptomology among nurses, factors such as sense of coherence, and social support emerged as protective resources. These protective factors have the potential to alleviate the mental health impacts of pandemic-related stressors among nurses.

The COVID-19 pandemic and its associated disease containment measures (DCMs) precipitated significant psychological distress among individuals across the globe. Generally, the public health crisis resulted in elevated levels of fear associated with contracting the virus and concerns about potential transmission to others (Church et al., 2022). This fear was exacerbated by heightened perceptions of vulnerability to disease, acknowledged as the subjective evaluation of the likelihood of infection (Yang et al., 2023). According to Church et al. (2022), individuals who perceive themselves as more vulnerable to infection are, in turn, more likely to experience heightened levels of fear, anxiety, and an overall sense of threat. Conversely, research suggests that perceptions of vulnerability to disease can promote self-protective behaviours such as hand sanitisation and mask-wearing, as well as enhance compliance with COVID-19 prevention measures, including stay-at-home directives and social distancing (Park et al., 2021; Sobkow et al., 2020; Yang et al., 2021). Nevertheless, increased levels of fear and elevated perceptions of vulnerability to disease may potentially compromise an individual’s sense of safety and control, resulting in adverse mental health outcomes as evidenced, for example, among nurses (Sakai et al., 2022), teachers (Pretorius et al., 2022), students (Kagee et al., 2024), dentists (Salehiniya et al., 2022), community-dwelling psychiatric patients (Ali et al., 2022) and pregnant women (Fan et al., 2022).

The inherently demanding nature of the nursing profession has consistently exposed these essential workers to heightened levels of job-related stress and subsequent distress (Roomaney et al., 2017), surpassing levels observed in colleagues in similar occupational domains, during comparable disease outbreaks (Brooks et al., 2018; Ricci-Cabello et al., 2020). Factors during the COVID-19 pandemic which had a significant impact on the mental health of nurses included, for example, the sudden escalation in severely ill patients, excessive working hours, an enhanced risk and fear of COVID-19 infection (Nie et al., 2020), the emotional toll of high mortality rates and witnessing expansive suffering, as well as inadequate access to personal protective equipment (PPE; Greene et al., 2021; Iddrisu et al., 2023; Levi-Belz and Zerach, 2022; Shanafelt et al., 2020). Furthermore, feelings of uncertainty, coupled with ongoing shifts in healthcare protocols, intensified stress among nursing professionals, while compliance to physical distancing mandates amplified experiences of social isolation, which is a critical factor protecting against stress (Karadaş and Duran, 2022). Moreover, poor mental health outcomes among nurses during the pandemic were compounded by the experience of moral distress stemming from difficult triage decisions, as well as contending with societal stigma associated with being perceived as a threat to others (Maben and Bridges, 2020).

The unique socio-economic and healthcare landscape in South Africa contributes an additional layer of complexity to the experiences of nurses, shaping their coping mechanisms and influencing their mental health outcomes. Nurses in South Africa, for example, not only contend with the unprecedented challenges posed by the COVID-19 pandemic but also with pre-existing challenges, such as high disease burdens, insufficient human capital, poor leadership, and major resource disparities (Harris et al., 2011; Kagee et al., 2011; Malakoane et al., 2020). Yet, despite the significant stressors associated with the pandemic, many nurses coped adeptly and demonstrated resilience. Differences in responses to stress among nurses likely point to the role of protective factors or generalised resistance resources (GRRs; Antonovsky, 1979, 1989).

GRRs, which form part of the salutogenic model of health (Antonovsky, 1979, 1989) are characteristics inherent within a person that helps them to better cope with stressors and contributes to their overall sense of coherence (Antonovsky, 1979, 1989). These GRRs may be influenced by (amongst other factors) one’s access to material resources (e.g. money), coping strategies, sense of coherence, social support, state of mind and preventative health orientation (see Horsburgh and Ferguson, 2000; Idan et al. Eriksso, 2017). In the current study, we examined sense of coherence, satisfaction with life and resilience as potential GRRs in the association between fear of COVID-19, social support, perceived vulnerability to disease and depression, respectively. Sense of Coherence (SOC) refers to ‘a generalised orientation towards the world which perceives it on a continuum, as comprehensible, manageable, and meaningful’ (Antonovsky, 1996: p. 15). Comprehensibility, which is the cognitive facet of SOC, entails intrinsic and contextual stimuli that are considered understandable, logical, and predictable. Manageability, the behavioural component of SOC, concerns the extent to which an individual perceives to have adequate resources to navigate daily stressors. Lastly, meaningfulness, as the emotional element of SOC, signifies the degree to which individuals perceive life challenges not only as obstacles, but as valuable pursuits that warrant attention and effort (Antonovsky, 1989). Taken together, SOC constitutes a pivotal element within the salutogenesis model, showcasing its profound impact as a promoter of mental health among nursing professionals. As evidenced by Masanotti et al. (2020) in a comprehensive systematic literature review, SOC emerges as a crucial health-promoting resource within the nursing community. More recently, Fei et al. (2023) found that SOC played a mediating role between workplace violence and burnout among Chinese nurses (*n* = 1190), while Hasimi et al. (2023) demonstrated that low SOC was a significant predictor of poor psychological outcomes among nursing students (*n* = 310) in Iran. According to Diener et al. (1985), satisfaction with life is defined as a holistic and cognitive evaluation of an individual’s overall life experience, which encompasses various domains and emphasises a subjective sense of wellbeing. Resilience, comparatively, refers to an individual’s ability to effectively adapt when confronted with adversity (Connor and Davidson, 2003). Satisfaction with life and resilience both serve as GRRs, which contribute to the enhancement of mental wellbeing. Indeed, studies have demonstrated that mental health outcomes are influenced by life satisfaction and resiliency factors. Otaghi et al. (2023), for example, found an inverse and significant relationship between life satisfaction and poor mental health outcomes among nurses (*n* = 160) in Iran. Similarly, Zborowska et al. (2021) found that burnout was significantly higher among nurses in Poland (*n* = 625) with reduced levels of life satisfaction. Likewise, when higher life satisfaction is reported, it functions as a source of resilience. Martins et al. (2022), for example, found that life satisfaction partially mediated the association between stress and personal burnout, depression and work-related burnout, and the association between anxiety and client-related burnout among Portuguese nurses (*n* = 379).

Several studies have also investigated the association between resilience and nurses’ mental health (e.g. Andersen et al., 2021; Lara-Cabrera et al., 2021). Lara-Cabrera et al. (2021), for example, found significant negative associations between resilience and reported levels of anxiety, depression and perceived stress among nurses (*n* = 214) in Spain. Similarly, Andersen et al. (2021) demonstrated a significant negative relationship between stress and resiliency among nurses (*n* = 328) in the United States. Studies have also examined resilience as a mediating factor. For example, studies demonstrate that resilience plays a partial mediating role in the connections between the mental health of nurses and pandemic fatigue (Labrague, 2021), as well as between emotional labour and symptoms of depression (Jung and Baek, 2020).

Recognising the essential role that nurses play within South Africa’s healthcare system, and the limited literature available on this vulnerable workforce amidst the COVID-19 pandemic within the country, it is necessary to understand the psychological underpinnings of their responses to the public health crisis. This understanding is vital for designing targeted interventions that address their immediate concerns and promote long-term mental wellbeing. The aims of this study were thus twofold namely: (i) to investigate the association between fear of COVID-19, social support, perceived vulnerability to disease and depression; and (ii) to determine the extent to which sense of coherence, satisfaction with life and resilience mediated these relationships. We postulated that there would be a positive association between fear of COVID-19, perceived vulnerability to disease and depression, as well as a negative association between social support and depression. In addition, we hypothesised that sense of coherence, satisfaction with life and resilience would mediate the relationship between fear of COVID-19, social support, perceived vulnerability to disease and depression. Figure 1 offers a diagram of the model followed.

**Figure 1.**
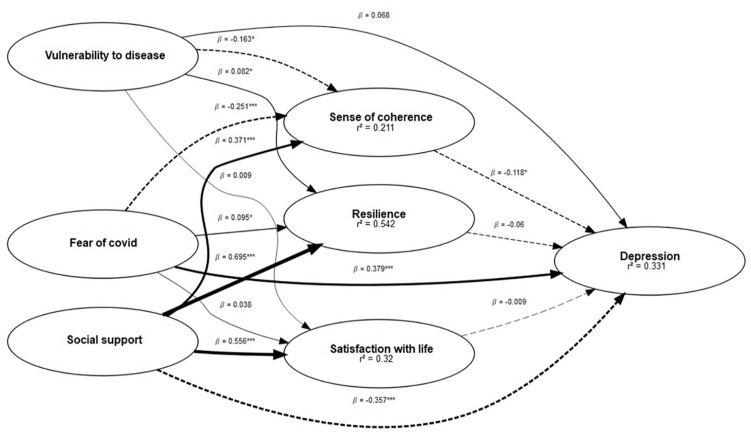
Structural equation model of the interrelationship between variables. Regression weights are standardised (***p < 0.001; **p < 0.01; *p < 0.05).

## Methods

### Participants and procedure

In this cross-sectional investigation, participants were a convenience sample of 264 nurses from four public hospitals situated in the Western Cape province of South Africa. The data were collected between April 2022 and May 2023. Recruitment occurred by means of notices displayed within the hospitals, inviting interested nurses to contact the research team via an allocated WhatsApp number. On contacting the team, participants were forwarded a link to an online consent form and questionnaire through WhatsApp. Participants were also able to complete hard copies of the questionnaire battery. Questionnaire booklets were handed out to interested participants at each of the hospitals. Completed booklets were dropped into a sealed dropbox in the break room and collected weekly by research assistants. Participation in the study was voluntary and anonymous. Notably, the sample consisted predominantly of female participants (82%) working in the government sector (76%). The average age of participants was 34 years (SD = 7.99).

### Instruments

Participants completed a short demographic questionnaire and a battery of self-report instruments as follows:

*Demographic data* were collected on age, gender, and which sector participants worked in.

*Satisfaction with Life* was measured using the Satisfaction with Life Scale (SWLS), which comprises five items rated on a 7-point Likert scale. A meta-analysis of 62 studies revealed a mean Cronbach’s alpha of 0.78, with some degree of variability (s = 0.09), with 95% confidence intervals (Vassar, 2007). Moreover, the 2-month test-retest correlation coefficient stood at 0.90. The scale’s reliability was affirmed among South African teachers (Pretorius et al., 2022) and students (Kagee et al., 2024). In this study, the SWLS demonstrated strong internal consistency, with a Cronbach’s alpha of 0.92.

*Sense of Coherence* was assessed on the 13-item Sense of Coherence (SOC) scale, where responses were provided on a 7-point Likert scale. This scale comprises three subscales: Meaningfulness (4 items), Comprehensibility (5 items), and Manageability (4 items). Prior research by Antonovsky (1993) and Söderhamn and Holmgren (2004) indicated Cronbach’s alpha values ranging between 0.79 and 0.95 for the 29-item version, and between 0.74 and 0.91 for the 13-item version. Each item on the scale involves a Likert-type response scale with seven gradations, contributing to a total score ranging from 13 (indicating low SOC) to 91 (representing the highest possible SOC). The scale’s reliability was affirmed among South African teachers (Padmanabhanunni et al., 2022a) and students (Kagee et al., 2024). Within our study, the internal consistency of the scale, as measured by Cronbach’s alpha, yielded a value of 0.67. *Resilience* was evaluated utilising the Connor-Davidson Resilience scale (CD-RISC), which comprises 25 items, each rated on a 5-point scale (ranging from 0 to 4). Participants indicated their feelings over the preceding month, with scores ranging from 0 to 100, where higher scores denote greater resilience. Connor and Davidson (2003) reported intraclass correlation and Cronbach’s alpha values exceeding 0.70. The scale’s psychometric properties were found to be acceptable among South African teachers (Padmanabhanunni et al., 2023) and students (Kagee et al., 2024). In this study, internal consistency, as gauged by Cronbach’s alpha, yielded a value of 0.95.

*Depression* was assessed with the Centre for Epidemiological Studies Depression Scale Revised (CESD-R), which is a widely utilised 20-item screening instrument (Eaton, 2001). This scale inquires about various depressive symptoms experienced in the past week. Aligned with the DSM-5, the CESD-R encompasses nine categories of major depressive symptoms, including dysphoria, anhedonia, appetite and sleep disturbance, cognitive impairment, guilt, fatigue, psychomotor agitation, and suicidality. Responses are rated on a scale: ‘not at all’ or ‘less than one day’ = 0; ‘1-2 days’ = 1; ‘3-4 days’ = 2; ‘5-7 days’ = 3; and ‘nearly every day for 2 weeks’ = 4. The total score is obtained by summing responses to the 20 questions, resulting in scores ranging from 0 (indicating no symptoms) to 60 (reflecting maximum symptom severity). The CESD-R demonstrates robust psychometric properties, including high internal consistency, and strong convergent and divergent validity with other measures (Van Dam and Earleywine, 2011). Previous studies with South African samples have effectively employed the CESD-R (Dreyer et al., 2019; Kagee et al., 2024; Mogambery et al., 2017). Within this study, the scale exhibited strong internal consistency, with a Cronbach’s alpha coefficient of 0.95. *The Perceived Social Support (PSS)* scale is a 10-item measure of the extent that participants perceive situations in their lives as stressful. It is scored on a 5-point scale that ranges from 0 (never) to 4 (very often). In South Africa, authors found the scale to have good internal consistency among a sample of South African university students (Kagee et al., 2024; Makhubela, 2022). The scale’s internal consistency as measured by Cronbach’s alpha in the current study was 0.97.

The *Fear of COVID-19 Scale (FCV-19S)* was employed to assess fear of COVID-19 (Ahorsu et al., 2022). Comprising seven items, the FCV-19S demonstrates a stable unidimensional structure with robust psychometric properties. Martínez-Lorca et al. (2020) reported acceptable internal consistency (α = 0.82) and test-retest reliability (intra-class correlatio*n* = 0.72) for the scale. Respondents are requested to express their level of agreement with the statements using a five-point Likert scale, ranging from ‘strongly disagree’ to ‘strongly agree’. The total score is computed by summing individual item scores, ranging from 7 to 35, with higher scores indicating greater fear of COVID-19. Notably, the scale’s reliability was found to be acceptable among South African teachers (Padmanabhanunni et al., 2022a) and students (Kagee et al., 2024). In the current study, Cronbach’s alpha coefficient yielded 0.94.

The 15-item *Perceived Vulnerability to Disease Scale (PVDS)* was used to assess participants’ perceived vulnerability to infectious disease. This measure encompasses two subscales: perceived infectability (consisting of 7 items) and germ aversion (comprising 8 items). Participants were prompted to indicate their agreement with each item on a seven-point scale, ranging from ‘strongly disagree’ to ‘strongly agree’. Approximately half of the items were reverse-scored (Duncan et al., 2009). Higher scores on the PVDS suggest a heightened perception of infectability, germ aversion, or overall vulnerability to disease. Notably, acceptable psychometric properties of the scale have been observed among South African teachers (Padmanabhanunni et al., 2022a,b) and students (Kagee et al., 2024). In our current sample, Cronbach’s alpha yielded 0.59. Although Churchill (1979) suggests a minimum Cronbach’s alpha of 0.6 as acceptable, the composite reliability for the PVDS was calculated at 0.83, surpassing Nunally’s (1978) recommended benchmark of 0.7. Thus, these findings indicate convergent validity within the measurement model; both the Cronbach’s alpha and composite reliability computed for the PVDS were considered acceptable.

### Data analysis

We used structural equation modelling (SEM) with the partial least squares approach (PLS-SEM) to analyse the data. For PVSD and SOC the composite scores of the sub-scales were used as manifest variables. For all the other scales, the actual items were used as manifest variables. The PLS-SEM was done using the ‘seminr’ package in R. The latent variables comprised sense of coherence, life satisfaction, resilience, depression, perceived fear of COVID-19, and perceived vulnerability to disease.

### Ethical considerations

This study obtained Ethics approval from the Health Research Ethics Committee at Stellenbosch University (ethics reference number: N21/05/012-COVID-19). Participation in the research was entirely voluntary and anonymous, with no incentives offered for involvement. Prior to engaging with the online survey, participants were required to provide their informed consent through the survey’s landing page. Additionally, participants were provided with contact information for free counselling services should they experience any distress resulting from their participation in the survey.

## Results

For assessing internal consistency, composite reliability values greater than 0.70 were deemed indicative of good reliability (Hair et al., 2019). In this study, composite reliability values ranged between 0.81 and 0.98. Convergent validity was evaluated through average variance extracted (AVE) values. All of the AVE values were above the guideline level of 0.5 (Mantsos et al., 2024). Discriminant validity was checked using heterotrait/monotrait ratios and were all found to be acceptable (Kline, 2023). All outer loadings of the manifest variables were statistically significant (*p* < 0.001) and ranged between 0.53 and 0.93 (>0.4). Variance inflation factors were checked for multicollinearity and were found to be acceptable. The intercorrelations, means and standard deviations are included in Tables 1 and 2.

**Table 1.** The direct effects of fear of COVID, perceived vulnerability to disease, and social support on depression (path coefficients are standardised).

Variables			Path co-efficient	95% lower	95% upper	*t* Statistic	*p* Value
FOC->SOC	FOC	SOC	−0.25	−0.38	−0.1	−3.57	<0.001
FOC->SWL	FOC	SWL	0.04	−0.09	0.18	0.56	0.573
FOC->Resilience	FOC	Resilience	0.09	0.01	0.18	2.09	0.037
FOC->Depression	FOC	Depression	0.4	0.29	0.51	7.27	<0.001
PVDS->SOC	PVDS	SOC	−0.16	−0.34	0.00	−1.94	0.053
PVDS->SWL	PVDS	SWL	0.01	−0.14	0.13	0.13	0.896
PVDS->Resilience	PVDS	Resilience	0.08	−0.01	0.18	1.81	0.071
PVDS->Depression	PVDS	Depression	0.08	−0.03	0.19	1.41	0.16
Social support->SOC	Social support	SOC	0.37	0.28	0.46	8.12	<0.001
Social support->SWL	Social support	SWL	0.56	0.45	0.65	10.71	<0.001
Social support->Resilience	Social support	Resilience	0.70	0.61	0.76	18.55	<0.001
Social support->Depression	Social support	Depression	−0.45	−0.54	−0.35	−9.08	<0.001
SOC->Depression	SOC	Depression	−0.12	−0.23	−0.02	−2.21	0.028
SWL->Depression	SWL	Depression	−0.01	−0.13	0.13	−0.13	0.895
Resilience->Depression	Resilience	Depression	−0.06	−0.21	0.10	−0.75	0.451

Note: FOC = fear of covid; PVDS = perceived vulnerability to disease; SOC = sense of coherence; SWL = satisfaction with life.

**Table 2.** Means, standard deviations and ranges of the variables.

Variable	Valid N	Mean	Minimum	Maximum	Std. Dev.
FOC	264	20.2	7.0	35.0	8.6
SWL	264	21.9	5.0	35.0	7.7
SOC	264	58.9	13.0	91.0	11.9
Resilience	264	27.2	1.0	40.0	9.3
Depression	264	20.5	0.0	70.0	15.7
Social support	264	61.0	12.0	84.0	19.1
PVDS	264	57.5	17.0	99.0	15.5

Note: FOC = fear of covid; PVDS = perceived vulnerability to disease; SOC = sense of coherence; SWL = satisfaction with life.

### Model direct path

Table 1 presents all significant specific direct effects. The structural equation model that was used to examine the direct and indirect effects of perceived vulnerability to disease, fear of COVID and social support on depression is presented in Figure 1. The direct effects resulting from the structural equation model in Figure 1 are presented in Table 1. As can be seen in Figure 1, we found several significant and non-significant relationships between variables. Fear of COVID-19 showed a significant positive relationship with depression (β = 0.4; *p* < 0.0001), while perceived vulnerability to disease did not significantly relate to depression (β = 0.08; *p* = 0.16). Social support (β = −0.45; *p* < 0.001) and sense of coherence (β = −0.12; *p* < 0.05) exhibited significant negative relationships with depression, whereas satisfaction with life (β = −0.01; *p* = 0.90) and resilience (β −0.06; *p* = 0.45) did not significantly correlate with depression. Moreover, fear of COVID-19 showed a non-significant relationship with satisfaction with life (β = 0.04; *p* = 0.57) but a significant negative relationship with sense of coherence (β = −0.25; *p* < 0.001). Perceived vulnerability to disease did not significantly associate with satisfaction with life (β = 0.01; *p* = 0.90) but exhibited a significant positive relationship with resilience (β = 0.09; *p* < 0.05). Social support demonstrated significant positive relationships with resilience (β = 0.70; *p* < 0.001), satisfaction with life (β = 0.56; *p* < 0.001), and sense of coherence (β = 0.37; *p* < 0.001). Additionally, a non-significant negative relationship was found between perceived vulnerability and sense of coherence (β = −0.16; *p* = 0.05). Furthermore, no significant relationships were observed between satisfaction with life (β = −0.01; *p* = 0.90), resilience (β = −0.06; *p* = 0.45), and perceived vulnerability to disease (β = 0.08; *p* = 0.16) with depression.

### Specific indirect effects

The indirect effects of fear of COVID-19, perceived vulnerability to disease and social support on depression are reported in Table 3. In relation to the mediating effects of sense of coherence, satisfaction with life and Resilience, non-significant relationships were found among all the variables except for the mediating relationship of sense of coherence between social support and depression (β = 0.05; *p* < 0.05).

**Table 3.** The indirect effects of perceived vulnerability to disease, fear of COVID, and social support on depression, including the mediators of sense of coherence, satisfaction with life, and resilience.

Variables	Path co-efficient	95% lower	95% upper	*t* Statistic	*p* Value
FOC->Resilience->Depression	−0.01	−0.03	0.01	0.64	0.52
FOC->SWL->Depression	0.00	−0.01	0.07	0.06	0.948
FOC->SOC->Depression	0.03	0.00	0.07	1.72	0.086
Social support->Resilience->Depression	−0.04	−0.15	0.07	0.76	0.45
Social support->SWL->Depression	0.00	−0.08	−0.01	0.13	0.897
Social support->SOC->Depression	−0.04	−0.09	0.01	2.00	0.046
PVDS->Resilience->Depression	0.00	−0.03	0.01	0.59	0.559
PVDS->SWL->Depression	0.00	−0.01	0.01	0.02	0.987
PVDS->SOC->Depression	0.02	0.00	0.05	1.49	0.137

Note: FOC = fear of covid; PVDS = perceived vulnerability to disease; SOC = sense of coherence; SWL = satisfaction with life.

## Discussion

This study was conducted after much of the COVID-19 pandemic had subsided. We sought to investigate the protective role of satisfaction with life, sense of coherence, and resilience in the relationship between depression, social support, fear of COVID-19, and perceived vulnerability to disease among nurses in South Africa. We found multiple significant relationships that underscore the salient role of protective factors in shaping mental wellbeing among nurses, particularly within the context of the stressors introduced by the COVID-19 pandemic. As expected, our study revealed a significant association between heightened levels of fear of COVID-19 and the manifestation of depression among the nursing cohort. This observation underscores that nurses who reported greater fear of COVID-19 were more likely to exhibit elevated depressive symptoms. This finding aligns with previous research findings (Alnazly et al., 2021; Chura et al., 2022). In the context of South Africa, factors such as high workloads, continual exposure to distressing situations, including patient suffering and mortality (Engelbrecht et al., 2021), and pre-existing challenges within the South African healthcare system (see, e.g. Harris et al., 2011; Kagee et al., 2011; Malakoane et al., 2020), likely exacerbated concerns about contagion among nurses. It is also plausible that nurses harboured fears not only for their own health but also for the wellbeing of their family and significant others, potentially contributing to feelings of vulnerability and subsequent depressive symptoms (Wu et al., 2020).

Similarly, our results show elevated levels of fear of COVID-19 significantly compromised nurses’ sense of coherence. Despite Antonovsky’s (1996) repeated emphasis on the pivotal role of sense of coherence in individuals’ coping mechanisms during adversity, our findings suggest that the heightened sense of threat posed by COVID-19 among South African nurses correlates with a reduction in their ability to comprehend the situation, feel confident in their coping capabilities, and derive meaning amidst uncertainty. This aligns with numerous studies across diverse populations which consistently indicate a negative correlation between sense of coherence and heightened levels of fear, stress, and anxiety (Dymecka et al., 2021; Erim et al., 2008). It is possible that the demanding nature of the nursing profession during the pandemic, characterised by prolonged working hours and heightened exposure to the virus, may have exacerbated burnout and contributed to the erosion of nurses’ sense of coherence. Furthermore, nurses experiencing intense fear may potentially resort to maladaptive coping strategies, such as substance use, gambling and self-harm, thereby further undermining their sense of coherence.

Conversely, consistent with our expectations, both sense of coherence and social support demonstrated significant negative associations with depression, suggesting their potential as protective resources. Indeed, the established literature underscores the protective function of sense of coherence against depression (Li et al., 2023; Masanotti et al., 2020). This protective mechanism stems from individuals with a robust sense of coherence being adept at mobilising appropriate resources to confront stressors and viewing challenging life events as surmountable hurdles, thereby diminishing their susceptibility to adverse mental health outcomes (Kulcar et al., 2023). Our findings align with prior research highlighting the mitigating influence of sense of coherence on depression among nurses (Hasimi et al., 2023; Masanotti et al., 2020). Similarly, social support has been established as a protective factor against depression (Gariépy et al., 2016). Consistent with existing literature, our study corroborates the protective role of social support against depression (e.g. Alnazly et al., 2021; Ortiz-Calvo et al., 2022; Zhan et al., 2022). Social support offers a means for individuals to navigate life’s challenges, satisfy their psychological needs, enhance internal stability, and bolster internal resources. The extant literature underscores the profound psychological distress experienced by healthcare workers (HCWs) during the pandemic, exacerbated by periods of social isolation from peers and reduced social interactions (Stubbs and Achat, 2022). In addition to living under the social restrictions experienced by the general public, HCWs in particular encounter heightened risks of virus exposure at work, fear of transmitting the virus to loved ones, enforced separation from family members to minimise transmission risks, societal stigma, and even instances of abuse or aggression from the public (Ananda-Rajah et al., 2020; Jang et al., 2021; Mehta et al., 2021). Consequently, promoting avenues for social support among nurses, particularly in times of crisis, emerges as a critical strategy to mitigate risks of depression. Notably, while fear of COVID-19 had a significant impact on depression and sense of coherence, it did not show a significant association with other indicators of mental wellbeing, such as satisfaction with life or resilience. This result may suggest that worries or fears related to COVID-19 may not necessarily substantially undermine or interfere with these protective factors. Additionally, it raises the possibility of some degree of adaptation to the fear over time. Our data, collected between April 2022 and May 2023 in South Africa, indicate that as time passed, nurses may have evaluated their fears and concerns regarding COVID-19 differently compared to their perceptions during the initial stages of the pandemic.

Our findings appear to be in contrast with other studies that found that depression was significantly negatively correlated with resilience (e.g. Lara-Cabrera et al., 2021; Yörük and Güler, 2021) and satisfaction with life among nurses (e.g. Martins et al., 2022; Otaghi et al., 2023). While the absence of significant relationships between resilience, satisfaction with life and depression among nurses in this study may seem counterintuitive based on theoretical expectations, several methodological, conceptual, and contextual factors may contribute to this result. Further research employing longitudinal designs, comprehensive measurement approaches, and consideration of mediating or moderating factors is warranted to better understand the complex interrelationships between these protective factors and depression among the South African nursing population. In this study, we found that sense of coherence mediated the link between social support and depression, consistent with existing research findings (Li et al., 2023). Nurses with a low sense of coherence, characterised by limited understanding of stressors, scarce coping resources, and a perception of life lacking purpose, may exhibit heightened susceptibility to depressive symptoms when they perceive limited available social support. Conversely, individuals with a well-developed sense of coherence are better equipped to leverage coping resources to navigate constraints in social support, potentially safeguarding them against depressive symptoms. Numerous studies consistently underscore the pivotal role of a well-developed sense of coherence as a mediator in the pathway to depression (Kulcar et al., 2023). Nevertheless, notably, Li et al. (2023) discovered that social support played a significant role in restoring an individual’s sense of coherence. It is thus plausible that sense of coherence represents a crucial psychological resource that can be fortified through social support, as proposed by Malinauskienė et al. (2009) and Pasek et al. (2017). The results of this study illuminate critical clinical implications for the mental health of nurses amid public health crises. Firstly, the robust correlation between heightened fear of COVID-19 and depressive symptomatology underscores the urgent need for interventions aimed at reducing fear. Secondly, the identification of sense of coherence and social support as significant protective factors calls for interventions focussing on improving nurses’ ability to comprehend, manage, and find meaning in public health emergency situations. Additionally, strengthening peer networks, counselling services, and organisational support structures should be a key intervention focus.

### Strengths and limitations

This study presents both strengths and limitations. Noteworthy strengths include its unique contribution to understanding a vulnerable workforce, essential in the context of the COVID-19 response. However, the study also encounters limitations that hinder the generalisability of its findings. Firstly, the cross-sectional data were collected from only four hospitals out of more than 600 in South Africa, with significant variations in resource accessibility among provinces and hospitals. Secondly, participants voluntarily chose to participate in the survey, introducing self-selection bias. Finally, the study relied on self-reported information, thereby restricting the capacity to indicate strong causal relationships.

## Conclusion

The COVID-19 pandemic has taken a psychological toll on many groups, not least health workers. Nurses in particular form the backbone of the public health system in South Africa and as such as a precious human resource. Enhancing wellbeing, sense of coherence and mental health among nurses will play an important role in reducing burnout, absenteeism, resignation, and emigration. To this extent the public health service in South Africa can be strengthened.

## Supplemental Material

sj-docx-1-hpq-10.1177_13591053241279000 – Supplemental material for Coping in crisis: The role of sense of coherence, life satisfaction, and resilience in the relationship between depression, social support, fear of COVID-19, and perceived vulnerability to disease among nurses in South Africa Supplemental material, sj-docx-1-hpq-10.1177_13591053241279000 for Coping in crisis: The role of sense of coherence, life satisfaction, and resilience in the relationship between depression, social support, fear of COVID-19, and perceived vulnerability to disease among nurses in South Africa by Bronwyne Coetzee, Phillipa Haine, Martin Kidd, Lindokuhle Shongwe, Marnus Janse Van Vuuren and Ashraf Kagee in Journal of Health Psychology
