# Supplementary material for: Coping in crisis: The role of sense of coherence, life satisfaction, and resilience in the relationship between depression, social support, fear of COVID-19, and perceived vulnerability to disease among nurses in South Africa
Source: J Health Psychol. 2024 Sep 30;30(10):2542–56. doi: 10.1177/13591053241279000 (PMC12979636; doi:10.1177/13591053241279000)
Supplement: sj-docx-1-hpq-10.1177_13591053241279000 – Supplemental material for Coping in crisis: The role of sense of coherence, life satisfaction, and resilience in the relationship between depression, social support, fear of COVID-19, and perceived vulnerability to disease among nurses in South Africa [file sj-docx-1-hpq-10.1177_13591053241279000.docx]

**Supplementary Material**

**Table 1**

Bivariate correlated among the variables

| **Variable 1** | **Variable 2** | **Pearson** | **Pearson p-val** | **n** |
| --- | --- | --- | --- | --- |
| FOC | SWL | 0.15 | 0.02 | 264 |
| FOC | SOC | -0.24 | <0.01 | 264 |
| FOC | Resilience | 0.26 | <0.01 | 264 |
| FOC | Depression | 0.35 | <0.01 | 264 |
| FOC | Social Support | 0.19 | <0.01 | 264 |
| FOC | PVDS | 0.39 | <0.01 | 264 |
| SWL | SOC | 0.33 | <0.01 | 264 |
| SWL | Resilience | 0.48 | <0.01 | 264 |
| SWL | Depression | -0.22 | <0.01 | 264 |
| SWL | Social Support | 0.56 | <0.01 | 264 |
| SWL | PVDS | 0.08 | 0.19 | 264 |
| SOC | Resilience | 0.24 | <0.01 | 264 |
| SOC | Depression | -0.35 | <0.01 | 264 |
| SOC | Social Support | 0.31 | <0.01 | 264 |
| SOC | PVDS | -0.22 | <0.01 | 264 |
| Resilience | Depression | -0.24 | <0.01 | 264 |
| Resilience | Social Support | 0.72 | <0.01 | 264 |
| Resilience | PVDS | 0.19 | <0.01 | 264 |
| Depression | Social Support | -0.36 | <0.01 | 264 |
| Depression | PVDS | 0.19 | <0.01 | 264 |
| Social Support | PVDS | 0.1 | 0.1 | 264 |

Note: FOC=Fear of Covid; PVDS=Perceived Vulnerability to Disease; SOC= Sense of Coherence; SWL=Satisfaction with Life
